# Uncovering regional variability in disturbance trends between parks and greater park ecosystems across Canada (1985–2015)

**DOI:** 10.1038/s41598-018-37265-4

**Published:** 2019-02-04

**Authors:** Douglas K. Bolton, Nicholas C. Coops, Txomin Hermosilla, Michael A. Wulder, Joanne C. White, Colin J. Ferster

**Affiliations:** 1 Integrated Remote Sensing Studio, Department of Forest Resources Management, University of British Columbia, 2424 Main Mall, Vancouver, BC V6T 1Z4 Canada; 2 Canadian Forest Service (Pacific Forestry Centre), Natural Resources Canada, 506 West Burnside Road, Victoria, BC V8Z 1M5 Canada; 3 Department of Geography, University of Victoria, PO BOX 1700 STN CSC, Victoria, BC V8W 2Y2 Canada

## Abstract

We assess the protective function of Canada’s parks and protected areas (PPAs) by analyzing three decades of stand-replacing disturbance derived from Landsat time series data (1985–2015). Specifically, we compared rates of wildfire and harvest within 1,415 PPAs against rates of disturbance in surrounding greater park ecosystems (GPEs). We found that disturbance rates in GPEs were significantly higher (p < 0.05) than in corresponding PPAs in southern managed forests (six of Canada’s 12 forested ecozones). Higher disturbance rates in GPEs were attributed to harvesting activities, as the area impacted by wildfire was not significantly different between GPEs and PPAs in any ecozone. The area burned within PPAs and corresponding GPEs was highly correlated (r = 0.90), whereas the area harvested was weakly correlated (r = 0.19). The average area burned in PPAs/GPEs below 55° N was low (0.05% yr^−1^) largely due to fire suppression aimed at protecting communities, timber, and recreational values, while the average burn rate was higher in northern PPAs/GPEs where fire suppression is uncommon (0.40% yr^−1^ in PPAs/GPEs above 55° N). Assessing regional variability in disturbance patterns and the pressures faced by PPAs can better inform policy and protection goals across Canada and the globe.

## Introduction

The past four decades have seen an increase in both the number and area of parks and protected areas (PPA) globally^1^. As of 2016, approximately 14.7% of the Earth’s land area is under some form of protection^2^. Globally, there has been progress towards the Aichi target of protecting 17% of the Earth’s terrestrial area^3^, with the target projected to be achieved by 2020^4^. Protected areas are critical indicators for achieving a number of Millennium Development Goals, such as ensuring environmental sustainability, integration of the principles of sustainable development into country policies and programmes, and reversing the loss of environmental resources and biological diversity^5^. The installation of representative and connected PPAs has been identified as a key means to harbour biodiversity and critical habitats^5^. However, increases in the area of PPAs globally contrasts with reports that biodiversity is in decline^6–8^. Understanding the extent, and effects, of pressures on the PPA network is a key driver for conservation science^9^.

**Figure 1:**
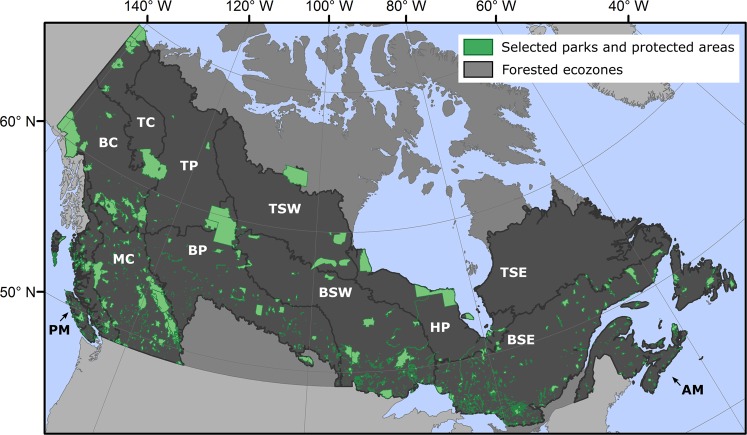
Map of the parks and protected areas (PPAs) selected for analysis across Canada**’**s forested ecozones. Ecozones are abbreviated: Atlantic Maritime (AM), Boreal Cordillera (BC), Boreal Plains (BP), Boreal Shield East (BSE), Boreal Shield West (BSW), Hudson Plains (HP), Montane Cordillera (MC), Pacific Maritime (PM), Taiga Cordillera (TC), Taiga Plains (TP), Taiga Shield East (TSE), and Taiga Shield West (TSW). Figure produced in ArcMap 10.5. PPA boundaries were obtained from http://www.ccea.org/download-carts-data/.

PPAs within Canada are administered by a variety of agencies including federal, provincial, territorial, First Nations, and regional governments^10^. By 2015, 10.6% (1.05 million km^2^) of Canada’s terrestrial area was recognised as protected, an increase from 9.8% in 2011 (Fig. 1)^11^. These PPAs function to preserve ecologically and culturally important areas and to promote economic development through tourism and recreation. In the context of a changing climate^12^, PPA are also desired to conserve ecological functions and evolutionary potential. Within a subset of PPAs, economic activity may include resource extraction, with various constraints and restrictions^13^. In Canada, the distribution of PPAs varies across the country, yet cover all 15 terrestrial ecozones to some degree, with the southern regions having a higher concentration of small protected areas while those in the north are larger and more widely dispersed^11,14^.

A common goal of park management agencies is the evaluation of PPA representation to ensure sufficient area is protected to offer preservation through the foreseeable future^13^. Andrew *et al*. (2011) used vegetation productivity gradients measured by satellite remote sensing to assess the representation (in terms of satellite-derived vegetation productivity) of PPAs within Canada. Representation varied by location and productivity, with large parks being biased towards less-productive areas (e.g., mountainous areas and northern latitudes), and smaller parks being located in more-productive areas where greater threats due to development are faced. Based upon Canada’s large area, low population, and lack of road access, it was found that some non-protected areas were functioning as *de facto* protected areas, facing little threat from development and functioning similar to natural or protected ecosystems^15^.

It is broadly recognized that PPAs are influenced by their surrounding area, because “national parks are part of larger ecosystems and must be managed in that context”^16^. PPAs are influenced by the surrounding area, as the PPAs themselves are part of larger ecosystems with significant ecological and socio-economic connection between PPAs and surrounding lands^17^. This concept was well established in the Greater Yellowstone Ecosystem (GYE)^17^. While originally defined as the range of the Yellowstone grizzly bear, definitions of the GYE have expanded over time to include the range and critical habitats for a number of species, with the aims of “maintaining all current plant and animal species, monitoring ecological processes, and integrating human economies with the other constraints”^18^. Likewise, plans for Canadian national parks often include examination of activities within the greater park regions that are based on detailed regional studies^19^. Soverel *et al*.^20^ studied national parks across Canada by sampling an equal area within the same ecozone as the park. More general definitions for park neighbourhoods can also be used. Palumbo *et al*.^21^ used 25-km buffers around protected areas in Africa to compare rates of wildfire occurrence. Although these definitions are not as specific as those developed for the management plans of individual parks, they do provide a general indication of how the area adjacent to the park compares to the area within the park and to areas further away.

Globally, the range of ecosystem disturbance that can impact the PPA and the surrounding areas is highly diverse and can include land use/land cover conversion for urbanisation or agriculture, wildfire, harvest, infestation by insects and other damaging agents, amongst others. Disturbances vary in their extent, duration, and severity^22^ and can also differ as a result of management practices that change at PPA boundaries, including fire exclusion, harvesting exclusion, or controlled burning programs^20^. Wildfire in particular is a critical natural process in many forested ecosystems^23^. In these dynamic systems, natural disturbance needs to be taken into account, and ensuring representation can be challenging because wildfires can be large in area, and occur infrequently^15^.

In the context of climate change, disturbance frequency (e.g., wildfire, insect infestation) is expected to increase^24,25^. PPAs serve as important benchmarks against which broader-scale changes (particularly anthropogenically caused changes) can be evaluated^26^. Consequently, baseline data characterizing historic rates of disturbances in PPAs relative to their greater park ecosystems (GPEs), as well as relative to their broader ecological context (e.g., ecozone), would be useful for future monitoring efforts. In a Canadian context, protection aims vary. In southern regions of the country with greater human populations, resource extraction opportunities, and rare-niche ecosystems, disturbance pressures on PPAs are greater. Additionally, the proximity of PPAs to communities and resources can lead to the exclusion of disturbances inside PPAs in populated areas. In more-northern Canadian environments, PPAs typically experience less anthropogenic disturbance pressure and are left to function more naturally. There is no single prescription for an appropriate level (or type) of disturbance that spans the range of ecosystem characteristics represented within Canada’s PPA system. Therefore, monitoring approaches that provide representation across time and space are desired^27^.

**Figure 2:**
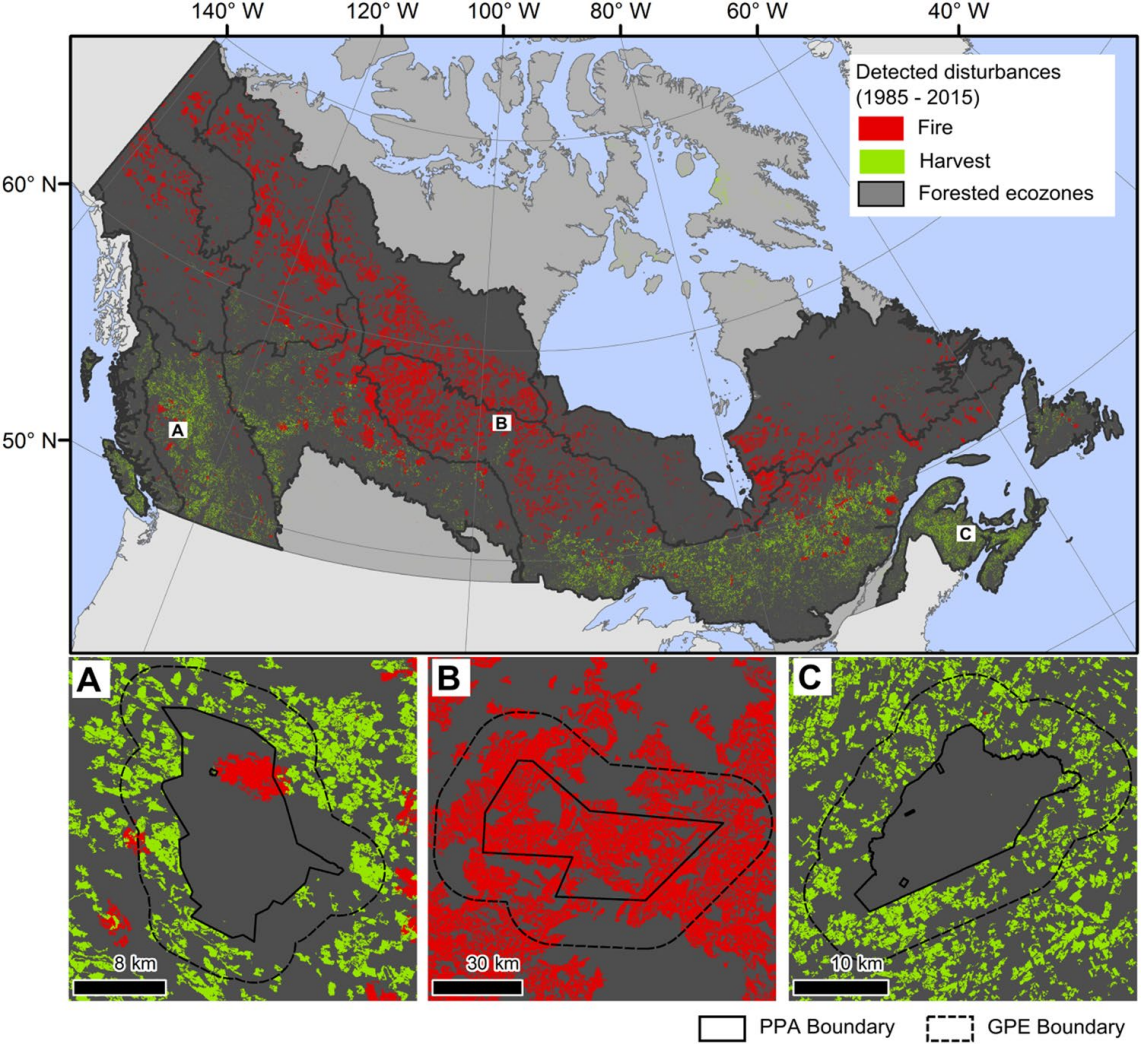
Areas detected as burned and harvested with Landsat data between 1985 and 2015 across Canada following the Composite2Change (C2C) approach. Panels A, B, C are examples of PPA and GPE boundaries for (**A**) Finger Tatuk Provincial Park, British Columbia **(B)** Amisk Park Reserve, Manitoba, and (**C**) Banoon Bog, New Brunswick. Figure produced in ArcMap 10.5. Disturbance data are available at https://opendata.nfis.org/mapserver/nfis-change_eng.html.

Recent advances in the availability and processing of remotely sensed data have enabled more-comprehensive and -accurate measures of forest change^28^, which in turn have facilitated retrospective national assessments of disturbance that are spatially explicit and spatially exhaustive, and uniquely generated from a single, consistent data source^29^. These data also allow for more-detailed and -extensive assessment of disturbance history in PPAs relative to their broader ecological context, and provide a comprehensive overview of the types and rates of disturbance within and surrounding PPAs^30^ (Fig. 2). In general, we expect to see the impact of large uncontrolled wildfire on more northern PPA and little disturbance present in PPA located in southern regions. We expect northern PPA to resemble greater park and ecosystem characteristics; whereas, we expect to see that more southern PPA differ from surrounding / encompassing areas related to both harvesting and to a lesser extent wildfire. Here, we demonstrate the capacity of advanced remotely sensed data analysis to examine disturbance patterns within and surrounding Canada’s PPA network through an assessment of 1,415 PPAs for a total area of over 44 million ha. To achieve this, we summarize a record of wildfire and harvest within PPAs, within the immediate neighbourhoods of PPAs (GPEs), and within the broader ecological context (ecozone) for each PPA.

## Results

**Figure 3:**
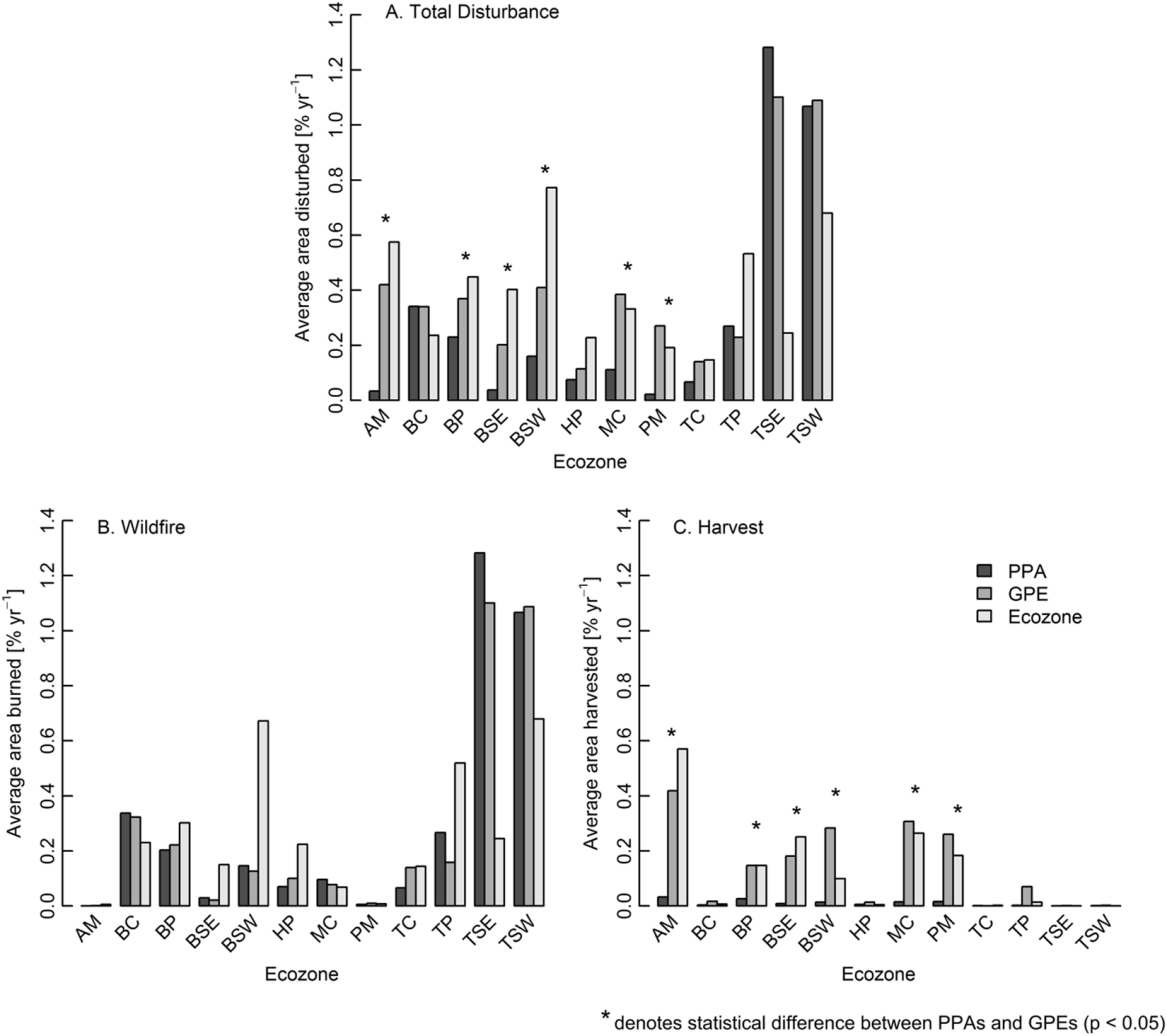
Average disturbance rates within PPAs, GPEs, and ecozones. Panel A displays total disturbance (i.e., wildfire and harvest combined) while panels B and C show wildfire and harvest separately. Asterisks denote if there was a significant difference (p < 0.05) between the area disturbed within PPAs and GPEs, using a two-sided paired t-test.

Figure 3 displays the average rate of disturbance within PPAs, GPEs, and ecozones for wildfire, silvicultural harvest, and combined wildfire and harvest (i.e., total disturbance). For harvest and wildfire combined (Fig. 3A), the average rate of disturbance was highest in PPAs within the Taiga Shield East (1.28% yr^−1^) and the Taiga Shield West (1.07% yr^−1^), and lowest within the Atlantic Maritime (<0.01% yr^−1^), Pacific Maritime (0.01% yr^−1^), and the Boreal Shield East (0.03% yr^−1^). The disturbance rate within GPEs was significantly higher (p < 0.05) than the disturbance rate within PPAs for the Atlantic Maritime, Boreal Plains, Boreal Shield East, Boreal Shield West, Montane Cordillera, and Pacific Maritime. When the disturbance rate was significantly higher in the GPEs, the GPEs tended to correspond more closely to the ecozone-level disturbance rate. Disturbance rates within PPAs were not significantly higher than GPE disturbance rates in any ecozone.

No significant differences in burn rates were found between PPAs and GPEs at the ecozone level (Fig. 3B). In general, the area burned within PPAs was more similar to the area burned within GPEs than to the area burned in the ecozones as a whole. In the Boreal Shield West, Hudson Plains, and Taiga Plains, the burn rate across the ecozone was markedly higher than the burn rate within the PPAs and GPEs, with the opposite trend observed in the Taiga Shield East and West.

Harvest rates were significantly higher in the GPEs than the PPAs in the Atlantic Maritime, Boreal Plains, Boreal Shield East, Boreal Shield West, Montane Cordillera, and Pacific Maritime (Fig. 3C). Ecozones that did not have a significant difference in harvesting between PPAs and GPEs had minimal harvest rates in both the PPAs and GPEs (<0.08% yr^−1^ harvested in both the PPAs and GPEs). Unlike area burned, the GPE harvest rates corresponded more closely to the ecozone as a whole than to the PPAs; the area harvested within PPAs amounted to less than 0.03% yr^−1^ over all ecozones. Average harvest rates in the GPEs were highest in the Atlantic Maritime (0.42% yr^−1^), followed by the Montane Cordillera (0.31% yr^−1^), the Boreal Shield West (0.28% yr^−1^), and the Pacific Maritime (0.26% yr^−1^).

**Figure 4:**
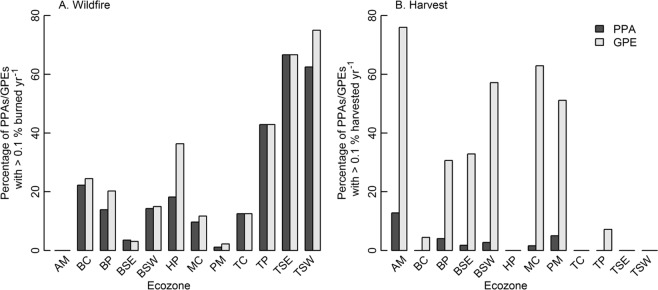
Percentage of PPAs and GPEs with disturbance (>0.1% disturbed yr^−1^) for (**A**) Wildfire and (**B**) Harvest from 1985–2015 by ecozone.

Figure 4 displays the percentage of PPAs and GPEs that had disturbances detected from 1985 to 2015 (i.e., percentage of PPAs/GPEs with >0.1% area disturbed yr^−1^). Over 60% of both PPAs and GPEs in the Taiga Shield East and West had fires detected, while less than 4% of PPAs and GPEs had fires detected in the Atlantic Maritime, Pacific Maritime, and Boreal Shield East. With the exception of the Hudson Plains, the percentage of PPAs with detected fires was similar to the percentage of GPEs with detected fires for all ecozones. Conversely, the percentage of GPEs with detected harvests was higher than in PPAs in all ecozones (Fig. 4B). The Atlantic Maritime had the highest percentage of GPEs with harvesting (76%), followed by the Montane Cordillera (63%), Boreal Shield West (57%), and Pacific Maritime (51%). On the contrary, no GPEs within the Hudson Plains, Taiga Cordillera, Taiga Shield East, or Taiga Shield West had an area that exhibited >0.1% harvested yr^−1^.

**Figure 5:**
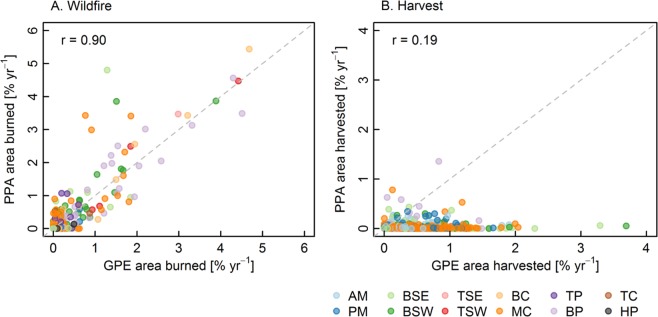
Scatterplot of disturbance rates within PPAs against disturbance rates in GPEs for (**A**) Wildfire and (**B**) Harvest. Correlation coefficients (r) are displayed.

The correspondence in disturbance rates between PPAs and GPEs was much stronger for area burned (r = 0.90) than for area harvested (r = 0.19, Fig. 5). When area burned was high within GPEs (e.g. >2% yr^−1^), the area burned within PPAs corresponded closely. Alternatively, high levels of harvesting within GPEs had no relationship to the level of harvesting detected within the PPAs. When large differences existed between the area burned in PPAs and GPEs, the area burned tended to be higher in the PPA than the GPE (e.g., PPAs >3% yr^−1^ burned while GPEs <2% yr^−1^).

**Figure 6:**
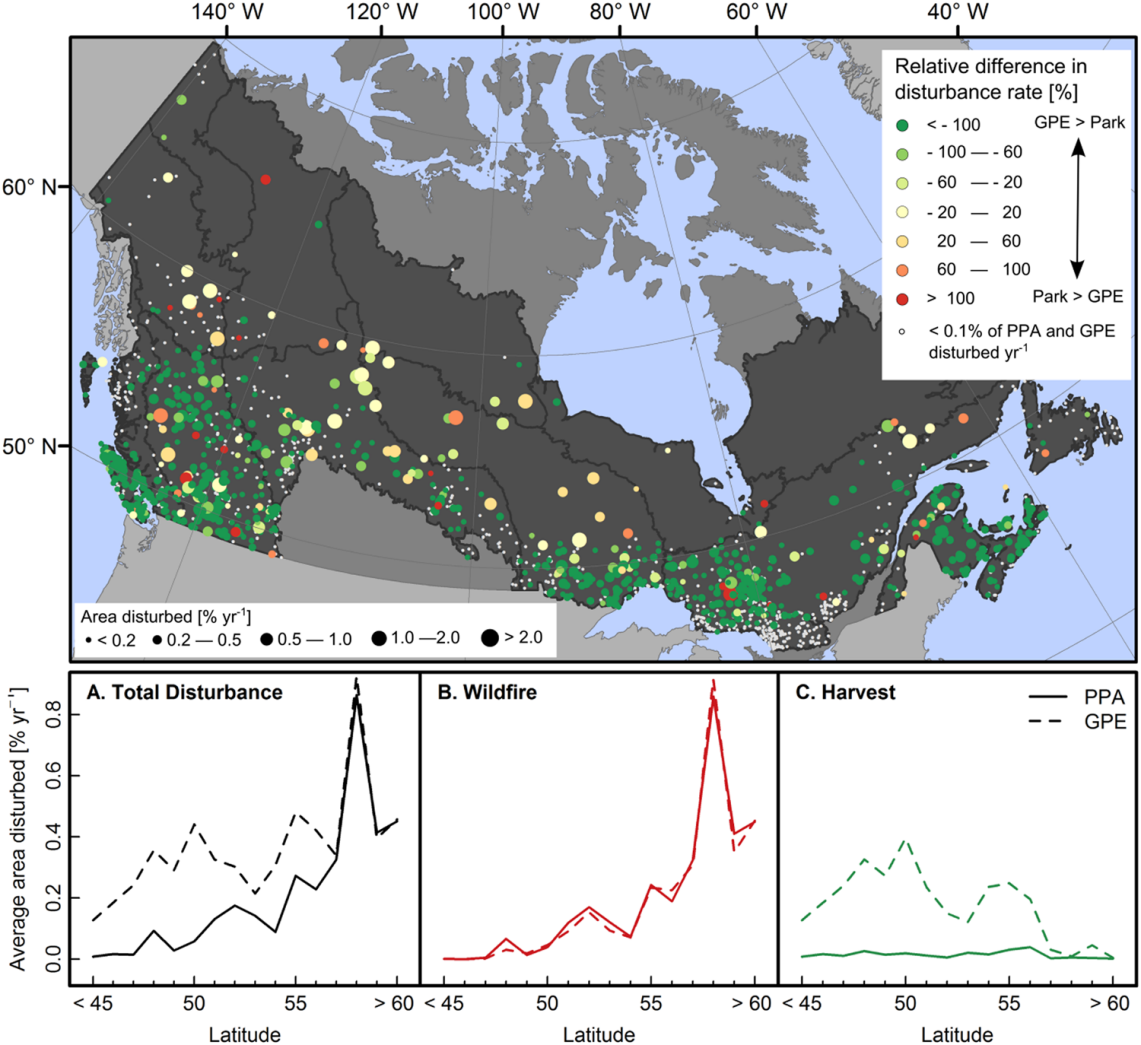
Relative differences in disturbance rates between PPAs and GPEs (fire and harvest combined). Point sizes correspond to the area of the PPA and GPE that was disturbed between 1985 and 2015. Panels A, B, and C display average disturbance rates for PPAs and GPEs by latitude for total disturbance, wildfire, and harvest, with PPAs/GPEs binned to the closest degree of latitude. Figure produced in ArcMap 10.5. PPA locations obtained from http://www.ccea.org/download-carts-data/.

Figure 6 displays the relative difference in disturbance rates between PPAs and GPEs for wildfire and harvest combined, as well as an assessment of disturbance rates by latitude. At low latitudes, disturbance rates tended to be higher within GPEs than PPAs, which was attributed to harvesting activities within the GPEs (Fig. 6C). At higher latitudes, where rates of harvesting were low (average harvest rate = 0.02% yr^−1^ in GPEs above 57° N), disturbance rates within GPEs tended to correspond more closely to disturbance rates within PPAs. PPA disturbance rates also tended to increase with latitude, which was driven by increases in the area burned (Fig. 6B). Specifically, the average area burned within PPAs/GPEs below 55° N was only 0.05% yr^−1^, but was 0.40% yr^−1^ for PPAs/GPEs above 55° N.

## Discussion

Remote sensing analyses have the capacity to monitor temporal and spatial disturbance patterns within, and across, the PPA network in Canada, allowing for an investigation of the complicated role PPA networks play in maintaining local disturbance regimes and ecological integrity^31,32^. Disturbance monitoring is a critical component of active management in PPAs. New approaches in remote sensing monitoring, as presented here, allow for a broad overview and comparison between PPAs, their GPEs, as well as the broader ecological unit in which the PPA belongs, which can provide insight to regional conservation policy goals that may not be apparent when evaluating individual PPAs, or single agencies^31^.

Of particular interest are the differences in area disturbed within PPAs and their GPEs. In the southern managed ecozones of Canada, GPEs had higher rates of disturbance than PPAs. Alternatively, in ecozones that are dominated by wildfire, rates of disturbance are more similar between PPAs and GPEs. While management agencies often allow fires to burn naturally within larger parks, allowing fires to burn in small parks in southern Canada is difficult, due to the risks imposed to neighboring communities (e.g., smoke, loss of property) and resources^33^. Further, in regions where rates of harvesting and development are high, small parks are often seen as harbours from change and disturbance, and fires negatively influence the aesthetics and recreational activities offered by parks, further motivating suppression activities and contributing to the larger discrepancies observed between PPA and GPE disturbance rates found in the more southern parks.

Harvest is the dominant disturbance type in the GPE networks of southern parks, where productivity is higher and timber harvesting is commercially viable. Wildfire is more prominent in the GPE networks of northern PPAs, where fire suppression is not a priority unless communities are at risk^33^. In areas of the landscape with very little anthropogenic influence, areas outside PPAs are “de facto PPAs” due to harsh climate and remoteness^10,15^. In northern PPAs, where harvesting is minimal or non-existent, there is little difference in annual disturbance rates between PPA and surrounding GPE (i.e., these PPA are characterized by low rates of human disturbance and high rates of natural disturbance). Large differences in total disturbance rates between GPEs and the broader ecozones (e.g., Boreal Shield West, the Taiga Plains, and the Taiga Shield East) suggest that existing PPAs are not distributed across the range of disturbance regimes found within the ecozone. However, as this trend was most prevalent in northern ecozones, where limited human access results in *de facto* protected areas, this is not of much concern.

Under more catastrophic wildfire conditions, fire suppression is less effective and PPA boundaries are somewhat irrelevant, which is likely why PPAs with extreme burn rates (e.g., >2% area burned yr^−1^) had similar rates of wildfire between PPAs and GPEs. In addition, as many of the PPAs with high rates of wildfire were in the north, where the value of forest resources within the GPE is low and fires are less aggressively suppressed or not suppressed, fires that start within PPAs will more likely be left to burn outside of the PPA boundary. Alternatively, in the south, fires outside of PPAs will be actively suppressed (if possible) to protect communities, infrastructure, timber and other ecosystem goods and services, resulting in greater rates of natural disturbance in PPAs relative to GPEs, which was observed for several parks in the Montane Cordillera and Boreal Shield ecozones (Fig. 5). In southern managed forests, fires within PPAs are often actively suppressed as well, in order to prevent fires from spreading to surrounding GPEs. Therefore, low rates of disturbance were found within southern PPAs and harvesting was the primary disturbance type in southern GPEs.

Through an investigation of the peer-reviewed literature, Nagendra^34^ found that PPAs in North America and Europe had lower rates of land clearing than PPAs in Asia, suggesting that the function and success of PPAs can vary regionally depending on the needs of the local community, the resources available to manage PPAs, and a range of additional factors. Further, Nagendra^34^ demonstrated that land clearing in North American and European PPAs was often for recreational purposes or for forest harvesting overseen by park managers, while the drivers in Africa, Asia, and Latin America were more complex and typically driven by local communities, which included cattle grazing, clearing for pastures, and firewood extraction. Despite these differences, some similarities do exist between the trends observed across Canada’s PPAs and those observed in the tropics. Similar to PPAs in Canada’s north, Joppa *et al*.^35^ found that many PPAs in the Amazon and Congo are *de facto* protected given their remoteness and inaccessibility, resulting in intact forest cover both within and surrounding these PPAs. Alternatively, in the South American Atlantic Coast and West Africa, where human populations are higher, Joppa *et al*.^35^ found distinct forest cover boundaries between PPAs and their surroundings, similar to the differences in disturbance rates we observed between PPAs and their surroundings in Canada’s southern, more populated, forests. These similarities demonstrate the important role that human population plays globally in determining the function of PPAs.

The establishment and maintenance of comprehensive, effectively managed, and ecologically representative systems of protected areas remains a critical goal designed to reduce the rate of global biodiversity loss^4,36^.While the goals of PPA networks are clear, there remains disagreement as to how best to manage PPAs^37^ and, more fundamentally, what the underlying purpose of PPA networks should be, with objectives of protection shifting from an initial focus on preservation with exclusion of all disturbances, towards a more-nuanced management model including a direct contribution to national development and poverty reduction^1^. In addition, it is now recognised that PPAs are influenced by their surrounding areas, as the PPAs themselves are part of a larger ecosystem with a significant ecological and socio-economic connection between the PPAs and surrounding lands^17^. As a result, there is growing recognition that PPAs now need to managed within this context^16^. Remote sensing based monitoring can provide critical information for management strategies to link disturbance within PPA boundaries to the greater ecosystems that they represent.

## Conclusions

Remote sensing and related land cover and change information offer opportunities to assess conditions and dynamics present across landscapes. PPA networks as mapped boundaries relate intended protection, while remotely sensed data can offer insights on realized protection. Wall-to-wall remote sensing-based assessments of forest disturbance provide a unique opportunity to assess both the protective function of PPAs as well as the disturbance pressures faced in the surrounding GPEs. Rates of disturbance were significantly different between PPAs and GPEs in half of the sampled ecozones, as harvesting activities in Canada’s managed ecosystems increase disturbance outside of PPAs, while fires are actively suppressed within PPAs/GPEs to protect resources, communities, and recreational opportunities. Alternatively, significant differences in disturbance rates between PPAs and GPEs were not found in Canada’s northern ecozones, where forests are not actively managed for timber and fires are often left to burn naturally—regardless of PPA boundaries. Parks located in regions with development pressures are managed to act as harbours of the habitat and biotic elements present, with less emphasis on maintaining disturbance regimes. Conversely, away from anthropogenic pressures, PPAs are generally left to function similarly to the containing ecosystem subject to the disturbance regimes that are present. As disturbance is a natural and necessary component of Canada’s forested ecosystems, maintaining disturbance regimes is a critical role of Canada’s PPAs. Balancing elements of natural processes with needs for protection of rare or threatened ecosystems is difficult and requires system-wide assessments. Assessments such as the one presented here offer information of value to ensure that PPAs maintain the disturbance characteristics of GPEs as well as larger ecosystems as a whole.

## Methods

### Study Area

Canada’s forested ecosystems occupy ~650 million ha, which represents ~ 65% of the land area of Canada^38^. The forested ecosystems of Canada consist of a mosaic of trees, shrubs, wetlands, and lakes, with the area of treed and other wooded land comprising ~347 million ha^39^. Canada’s forests occupy a wide range of climatic, topographic, and soil conditions^40^. The Ecological Stratification Working Group (1995) divided the Canadian landbase into 15 ecozones based on distinct biotic and abiotic features within an ecological unit, including climate, physiology, and vegetation. This study focuses on the forested ecozones of Canada (Fig. 1). As the Taiga and Boreal Shield ecozones are large and span a wide range of ecosystem and climatic conditions, these ecozones were split into east and west components^41^, resulting in a total of 12 forested ecozones.

Much of Canada’s northern forests are *de facto* protected, as these forests are remote, of low productivity, and difficult to access^15^, resulting in forested ecosystems that are dominated by natural disturbance^42^. The frequency of wildfire, the dominant natural disturbance agent in Canada’s forests^29^, generally increases from east to west across Canada, as conditions in the west are drier and the probability of lightning strikes is higher, with fire frequency varying from several decades to several centuries^43,44^. Much of Canada’s northern forests are *de facto* protected; whereas, within accessible, higher productivity, southern regions forests are actively managed for timber^45^. Looking forward, the lessons from this research offer some insight for incorporating the role of disturbance when expanding a PPA network. At present, there is interest in further expanding Canada’s PPA as described in the Federal Sustainable Development Strategy to meet the 17% area target by 2020^46^ [p. 48] agreed to under the Convention on Biological Diversity and related Aichi target^3^

### Data

#### Parks and protected areas

Spatial boundaries of Canadian PPAs were obtained from the Conservation Areas Reporting and Tracking System (CARTS), 2016 version (http://www.ccea.org/download-carts-data/). These data provide seamless coverage of PPAs in Canada, including IUCN classification. IUCN categories represent different levels of protection and different functions for PPAs. Categories Ia-IV can be considered strictly protected, while categories V and VI represent levels of protection that include limited anthropogenic disturbances^47^. Parks were included in the study if they were located within a forested ecozone, were at least 100 ha in size and assigned an IUCN classification of Ia, Ib, II, or IV within the CARTS database. Categories V and VI were not considered as the focus was on strictly protected PPAs, while category III was removed as these represent national monuments, such as landforms and geological features. As inter-annual variability in disturbance rates can be high within PPAs and GPEs, and a single year may be unrepresentative of longer-term trends, only PPAs that had been established for at least 10 years were analyzed (i.e., established by 2006). Table 1 lists the number of PPAs sampled by ecozone, while Fig. 1 maps the distribution of these parks.

**Table 1:** Number and area of PPAs analyzed by ecozone.

Ecozone	n	Min. size [ha]	Max size [ha]	Mean size [ha]
Atlantic Maritime (AM)	125	105	96,835	5,915
Boreal Cordillera (BC)	45	153	1,893,259	139,949
Boreal Plains (BP)	173	100	330,817	13,868
Boreal Shield E. (BSE)	453	103	394,036	10,679
Boreal Shield W. (BSW)	147	109	723,905	26,607
Hudson Plains (HP)	11	705	2,036,214	310,543
Montane Cordillera (MC)	248	101	988,388	30,299
Pacific Maritime (PM)	180	100	297,390	11,468
Taiga Cordillera (TC)	8	3,620	891,226	283,853
Taiga Plains (TP)	14	113	3,952,402	522,561
Taiga Shield E. (TSE)	3	6,751	33,376	21,974
Taiga Shield W. (TSW)	8	6,530	1,694,510	411,587
Canada-wide	1,415	100	3,952,402	31,184

#### Landsat-derived disturbance data

To quantify area burned and harvested across the forested ecozones of Canada, we used Landsat-derived disturbance information produced by Hermosilla *et al*.^48^ at 30 m spatial resolution (Fig. 2). Hermosilla *et al*. (2015) utilised Landsat TM and ETM + data to provide systematic, spatially and temporally complete, consistent, and repeatable classification of disturbance events across the forested regions of Canada. The end-to-end approach, called Composite2Change (C2C), applies spectral trend analysis to annual, cloud-free, surface reflectance, pixel-based image composites enabling detection of vegetation changes, characterization based on temporal, spectral, and geometrical properties (see^28^ for details), and attributing these change events to the most likely disturbance category, with a focus on fire and harvesting^49^. Independent assessment (see^49^ for details) confirms the changes are accurately detected both spatially (overall accuracy = 90%) and temporally (89% of the changes detected the correct occurrence year). Change events are also successfully attributed (87%) to the correct disturbance agent, with higher accuracy for stand-replacing fire and harvest events^48^. The Landsat-derived disturbance data is available from https://opendata.nfis.org/mapserver/nfis-change_eng.html.

### Defining Greater Park Ecosystems (GPE)

Each PPA was matched with a representative GPE by sampling 30-m pixels immediately adjacent to the PPA. Specifically, a search window was iteratively increased around each PPA until an area twice the size of the PPA was sampled. Pixels within and surrounding each PPA were first masked to remove water and agricultural lands using a Landsat-derived water mask^50^ and an agriculture mask provided by Agri-Foods Canada (http://www.agr.gc.ca). Further, to ensure that GPEs did not fall within other protected areas, pixels falling within other PPAs were masked. Once an area twice the size of the PPA was identified, 30 m pixels were randomly sampled within the GPE equal to the area of pixels within the PPA (Figure 2A–C).

### Comparing disturbance between PPAs, GPEs, and ecozones

The total area burned and harvested from 1985 to 2015 was summed for each PPA and GPE. The summed area was divided by the total land area of the corresponding PPA and GPE, as well as by the number of years analyzed, to derive estimates of percent area disturbed per year. Water and agriculture pixels were first removed prior to calculating the land area of each PPA and GPE. Further, PPA establishment dates, which were included in the CARTS dataset, were used to ensure that each PPA and GPE was only assessed for the time period of protection if the PPA was established after 1985. Each PPA was assigned to its corresponding ecozone, allowing ecozone-level summaries to be calculated for PPAs and GPEs. PPAs that crossed an ecozone boundary were assigned to the ecozone containing the centroid of the PPA. Ecozone-level estimates of area disturbed were derived by summing the total area burned and harvested across each entire ecozone during the study period (1985–2015). Similarly to the PPA and GPE estimates, water and agricultural pixels were first removed prior to calculating the land area of each ecozone.

Pearson’s correlation coefficients (r) were calculated between the percent area disturbed within PPAs and GPEs for both wildfire and harvests to assess the relationship between disturbance inside and outside of PPAs. Two-sided paired t-tests were conducted to determine if the percent area disturbed within PPAs was significantly different from the area disturbed within GPEs (α = 0.05). The paired t-tests were conducted for each ecozone. All statistical analyses were conducted in the R software package^51^

Additionally, the relative difference between PPA and GPE disturbance rates were calculated using the following equation:

$${Relative}\,{Difference}=100\ast \frac{PP{A}_{D}-GP{E}_{D}}{\frac{PP{A}_{D}+GP{E}_{D}}{2}}$$

where PPA_D_ and GPE_D_ represent the rates of disturbance for each PPA and GPE during the study period.

## Data Availability

The Landsat-derived disturbance data are available from https://opendata.nfis.org/mapserver/nfis-change_eng.html. Park and Protected Area boundaries are available from http://www.ccea.org/download-carts-data/.
